# Road to Efficient Health Maintenance Via the Hospitals

**Published:** 1919-08-16

**Authors:** 


### MINISTERS OF HEALTH.

### II. Road to Efficient Health Maintenance via the Hospitals.

There are considerations of paramount importance why the nursing profession should establish an irrefutable claim to perform all, or practically all, the women's work of the Ministry of Health. When these are realised there is little room for doubt that the policy of hospital committees and matrons will be framed to meet the national demand.

Let it not be forgotten that the Ministry of Health was formed in response to a national call. There are scores of Acts in the Statute-book useful and necessary, which the nation never considered prior to their enactment. In such cases Government anticipated the people. The Ministry of Health is the result of a great public awakening. It has become clear to the least informed that the national weal was being sapped by ignorance, carelessness, and neglect. In considering matters of this kind it is desirable to get down to bed-rock. What is in demand is efficiency. The supply is lacking, and those who are available to act as health visitors are very far from being the best possible. They are uneducated and untrained in the essentials, although doubtless willing and anxious to do their best. It is scarcely necessary to enlarge on this subject, because it is freely admitted by the highest and most responsible health authorities that a trained nurse who has specialised in this department of science is not alone the best possible, but is the only really competent worker. In her sphere she corresponds with a doctor holding a public health diploma.

### The Financial Question.

In rural districts the district nurse who is fully trained and possesses a midwifery qualification is an asset whose worth cannot be measured in money. Very often she is a cyclist and incurs the minimum of expense in attending to her patients, and, whether officially recognised or not, she is the health visitor of the district. It is extremely probable that while the new Department is, so to speak, finding its feet, she will become the official health visitor. But in the course of time as vacancies occur, and when a supply of women holding health diplomas are available, if the nurse is unqualified it may happen that in this branch she will be supplanted, and two people appointed to perform duties which really overlap, thus causing needless expense without added efficiency. At present the district nurse is underpaid. Her appointment as health visitor would be valuable to herself, and at the same time an economy to the community.

### The Hospital Point of View.

It may be pointed out that these arguments do not concern the training-schools, that hospitals are not established to promote the interests of the nurse, and that their functions are purely local. A big London hospital might with considerable show of reason disown all responsibility for the health of the inhabitants of Connemara or the Orkneys. It cannot be denied, however, that they are directly and intimately concerned with the efficiency of their own nursing staff. From this point of view I have heard it seriously advanced that if hospitals were to pay their nurses high salaries and reduce their hours they would remain as fixtures until age or infirmity caused them to retire. There would be nothing to entice them to other fields of employment, and there would be a lack, in consequence, of "new blood." I am not concerned with the ethics of this argument. Ethics were not being discussed, but merely a possible consequence of economic betterment. What I desire to point out is that if a new sphere of employment offers itself and is availed of by trained nurses, the flow of candidates is bound to increase and their social and educational standard to rise. There will be a continual exodus from the hospitals. Those who prefer the nursing side will remain, and for those whose taste is in the other direction there will be an abundance of suitable openings. In a word, the road to efficient health maintenance is *via* the hospitals. And at present the hospitals are unequipped.

Now what will happen if the hospitals pass this opportunity unnoticed, and fail to adapt their training machinery to the national necessities? Obviously the quality and quantity of candidates for training will decline, and young women of talent who would otherwise become probationers will find it more profitable and attractive to take out a course of instruction in one of the women's colleges. They are doing so even now.

### The Shadow and the Substance.

There has been a great deal of pursuing after the shadow. This is the substance. Never were truer words spoken than that there is no magic in registration. If and when it comes it should no doubt fulfil a useful purpose, but like soft words it will butter no parsnips in this generation at all events. The canker of nursing is economic, and if hospital matrons and hospital committees have the wit there is nothing to prevent nurses from capturing a monopoly of entry by this new and wide open door to remunerative and extremely useful and interesting employment. By so doing every hospital worker from matron to pupil must automatically benefit. Such benefit, moreover, will be more than material. It will, in addition, be moral and spiritual. The outlook and service of the nurse will be wider and her sphere of usefulness will be more intimately associated with the progress of the nation.

But there is no time to lose. The national clamour for better conditions of health is imperative, and if the nursing profession fails to respond with promptitude another set of people will come forward and capture the prizes. IERNE.

